# Positioning Students for Success: 2024 Student Paper Contest Winners, Student Committee Research Skills-Building, and Release of 2025 Call for Papers

**DOI:** 10.5888/pcd21.240504

**Published:** 2024-12-12

**Authors:** Leonard Jack

**Affiliations:** 1 Preventing Chronic Disease, Office of the Director, National Center for Chronic Disease Prevention and Health Promotion, Centers for Disease Control and Prevention, Atlanta, Georgia

*Preventing Chronic Disease* celebrated its 20th anniversary this year. In 2011, the journal initiated its first Student Paper Contest in which students from around the world submit research papers to the journal for consideration. In our ongoing commitment to mentoring future public health professionals, the journal has guided students in developing their scholarly writing skills, with the goal of helping them become established contributors to the public health literature. We are tremendously proud to have served over the past decade as a place for students to consistently develop their expertise in peer-reviewed publication. The journal offers rare opportunities for students to learn from our leadership and its rich pool of scholarly volunteers — associate editors, peer reviewers, and others — as well as from mentors at their respective institutions. Since launching the Student Paper Contest 13 years ago, the journal has welcomed submissions at all levels, ranging from high school and undergraduate students to masters, doctoral, and postdoctoral candidates. Regardless of whether papers are accepted for publication, PCD provides students with extensive feedback helpful in identifying areas for improvement that can facilitate conversations between students and their mentors and assist them in future publishing efforts.

## Goals and Submission Requirements

Eligibility requirements for the journal’s Student Paper Contest are refined annually but in general remain consistent to their original charge (1). Students submitting papers for consideration are currently enrolled in or have in the last 12 months completed their degrees and programs, high school diplomas, undergraduate and graduate degrees, medical residencies, or postdoctoral fellowships. For the contest, PCD considers only one of two types of papers: original research and GIS (Geographic Information Systems) Snapshots. The primary goals of the Student Paper Contest are in the following 5 areas:

- Provide students with an opportunity to become familiar with a journal’s manuscript submission requirements and peer-review process
- Assist students in connecting their knowledge and training on conducting quality research according to a journal’s publication expectations
- Develop students’ research and scientific writing skills to become producers of knowledge rather than just consumers of knowledge
- Provide students with an opportunity to become a first author on a peer-reviewed article
- Promote supportive, respectful, and mutually beneficial student–mentor relationships that strengthen students’ ability to generate and submit scholarly manuscripts through their professional careers

## 2024 Winners and Submissions

PCD received 20 student research papers for our 2024 contest. After careful internal review, 15 of these underwent peer review. Nine of the 15 were accepted for publication and appear in the 2024 Student Research Paper Collection. Student authors continue to demonstrate their expanding interest in timely public health topics that include the cost barrier of medications in a student-run free clinic, WIC (Special Supplemental Nutrition Program for Women, Infants, and Children) benefits for purchase of fruits and vegetables and food security, colorectal cancer knowledge and screening among men, the use of geospatial hot spots and cold spots in cancer-related disparities in the US, factors associated with access to mental health services among children in the US, lifestyle intervention program for Spanish-speaking immigrants without health insurance, trends in gestational weight gain and prepregnancy obesity, and exploring the evidence of outpatient follow-up visits to reduce readmissions for cardiovascular health conditions.

After careful review, PCD did not select winners in the undergraduate, master’s, and postdoctoral categories this year. We are, however, pleased to announce the winners of the 2024 Student Paper Contest in the high school and doctoral categories.

In the journal’s high school category, Meng and Wiznitzer, authors of “Factors Associated With Not Receiving Mental Health Services Among Children With a Mental Disorder in Early Childhood in the United States, 2021–2022,” reported findings about a strong link between health care factors and not receiving mental health services among children with a mental disorder in early childhood (2). The authors concluded that without timely treatment in early childhood, mental health disorders can impair children’s learning abilities and relationships with others and may contribute to lifelong complications.

Guo and colleagues produced the winning paper in the doctoral category: “Geospatial Hot Spots and Cold Spots in US Cancer Disparities and Associated Risk Factors, 2004–2008 and 2014–2018” (3). This paper identified factors with the strongest influence on creating hot spots and cold spots, including unemployment, preventable hospital stays, mammography screening, and high school education. Authors highlighted the need for targeted interventions and policies that address limited access to health care and its associated risk factors.

We congratulate all student winners and students of papers that were not accepted for publication. Over the years, PCD has heard from mentors that their students have benefited from this real-world scholarly writing opportunity. Our commitment to increasing the capacity of students to generate quality peer-reviewed submissions will continue to expand over the coming years.

## 2025 Call for Student Papers

Moving forward, PCD will offer 2 student paper opportunities. First, we will continue to welcome students’ interest in submitting research papers for the Student Paper Contest. We are interested in publishing research papers on the prevention, screening, and surveillance of chronic diseases and related population-based interventions, including but not limited to arthritis, asthma, cancer, depression, diabetes, obesity, cardiovascular disease, and COVID-19. Students are also invited to submit papers in response to our new essay submission call for papers, “Students Have Their Say: Novel Approaches and Solutions to Current and Emerging Public Health Problems.” This essay submission opportunity allows students to identify and discuss new approaches to persistent or emerging public health challenges. We believe that students are in a unique position to offer novel ideas and share fresh perspectives, and we want them to have their say.

To obtain detailed information about submission requirements for both opportunities, students, mentors, and readers are encouraged to visit our Calls for Papers page. Interested students are also encouraged to visit our Author’s Corner for important information on how to develop a manuscript and associated tables or figures. Both of our Student Paper contest (original research or GIS Snapshots only) and student essay contest (essay only) are due by 5:00 pm EST on Monday, March 24, 2025.

## Student Scientific Writing and Review Committee

Last year, PCD expanded its commitment to building the next generation of public health researchers by establishing its inaugural Student Scientific Writing and Review Training Committee. Last year’s students completed an extensive training course and received our certificate of completion. Training is well under way for the second round of student appointees from across the country. Students appointed to the 2024 student committee, upon completion of their near year-long appointment, will get exposure to the following skills and abilities:

- Understanding the purpose and function of a respected peer-reviewed journal
- Understanding submission requirements for specific article types offered by a peer-reviewed journal (eg, format, word count, headings, manuscript style)
- Using the journal’s electronic management tracking system to submit an article
- Becoming familiar with the peer-review process and how to appropriately respond to comments and suggestions from the journal
- Recognizing key components of a scientific research publication (quantitative, qualitative, or mixed methods)
- Understanding how to appraise the quality of each component of a publication
- Assessing strengths and weaknesses of an article’s methods, statistical analyses, data reporting, findings, and conclusions
- Applying skills developed from critiquing articles as a reviewer to generate quality papers as an author
- Writing strong introductions (ie, rationale), research questions, methods, findings, and conclusions
- Conducting literature reviews and creating bibliographies on topics of interests
- Working collaboratively with others within and outside one’s area(s) of interest and expertise to address complex public health challenges and opportunities
- Learning to distinguish between the lay writing and scholarly writing necessary for consideration by a peer-reviewed journal

We look forward to hearing great things about students benefiting from the many learning opportunities the journal offers. Please join us in congratulating students who are seeking these opportunities and using lessons learned in their future academic preparation and real-world work experiences.
